# Hair-follicle-associated pluripotent stem cells derived from cryopreserved intact human hair follicles sustain multilineage differentiation potential

**DOI:** 10.1038/s41598-019-45740-9

**Published:** 2019-06-27

**Authors:** Koya Obara, Natsuko Tohgi, Sumiyuki Mii, Yuko Hamada, Nobuko Arakawa, Ryoichi Aki, Shree Ram Singh, Robert M. Hoffman, Yasuyuki Amoh

**Affiliations:** 1 Department of Dermatology, Kitasato University School of Medicine, Minami Ward, Sagamihara, 252-0374 Japan; 2 Basic Research Laboratory, National Cancer Institute, Frederick, MD 21702 USA; 3 AntiCancer, Inc., 7917 Ostrow Street, San Diego, CA 92111 USA; 4 Department of Surgery, University of California, San Diego, CA 92103 USA

**Keywords:** Adult stem cells, Cell growth

## Abstract

The bulge area of the hair follicle contains hair-follicle-associated pluripotent (HAP) stem cells. Here, we present effective cryopreservation procedures of the human hair follicle that preserve the differentiation potential of HAP stem cells. Whole hair follicles isolated from human scalp were cryopreserved by a slow-rate cooling medium and stored in liquid nitrogen. A careful thawing method was used to collect the upper parts of the human hair follicles which were cultured for four weeks in a Dulbecco’s Modified Eagle’s Medium with fetal bovine serum (FBS). Proliferating hair follicle cells were then shifted to DMEM/Ham’s Nutrient Mixture F-12 medium without FBS and allowed to grow for one week. These proliferating cells were able to produce HAP stem cell colonies with multilineage differentiation capacity. They produced keratinocytes, smooth muscle cells, cardiac muscle cells, neurons and glial cells. Interestingly, these cryopreserved hair follicles produced pluripotent HAP stem cell colonies similar to fresh follicles. These findings suggest that the cryopreserved whole human hair follicle preserves the ability to produce HAP stem cells, which will enable any individual to preserve a bank of these stem cells for personalized regenerative medicine.

## Introduction

Hair-follicle bulge stem cells were originally shown to have the capacity to form hair-follicle cells, sebaceous-gland basal cells, and epidermis^1–5^. In addition to normal tissues homeostasis, these stem cells are involved in the regeneration of hair follicles and acute epithelial wound repair^6,7^. Subsequently, we discovered nestin-expressing stem cells in the bulge area of the hair follicle^8,9^. We found that nestin-expressing stem cells from both mouse and human have multilineage differentiation capacity that could produce neurons and other cell types^10–13^. We termed these nestin-expressing stem cells hair-follicle-associated pluripotent (HAP) stem cells. Previously, we demonstrated that isoproterenol and hypoxia can enhance the HAP stem cells to form cardiac muscle cells^13,14^. Further, it was shown that HAP stem cells from both mouse and human can fully repair the severed sciatic nerve and spinal cord of mice^15–17^ and showed homing capacity in experimental cortical dysplasia^18^.

Using a slow-rate cooling method, we previously demonstrated that cryopreserved whole-mouse hair follicles were able to maintain the pluripotency of HAP stem cells^19^. In the present study, we established effective cryopreservation procedures of the whole human hair follicle by slow-rate cooling and storage in liquid nitrogen, which preserved the multilineage-differentiation capacity of human HAP stem cells.

## Materials and Methods

### Isolation of human scalp skin samples

The human HAP stem cells were isolated from specimens obtained with surgery of normal human scalp from five patients^20^. The age of five patients ranged from 32 to 72 years. All patients had given informed consent to Kitasato University, School of Medicine to perform this study. This study was done with the approval of the Kitasato University Medical Ethics Organization. Experiments in the present study were performed per the Declaration of Helsinki guidelines and in agreement with national regulations for the experimental use of human material^20^.

### Cryopreservation of human whole hair follicle

**Figure 1:**
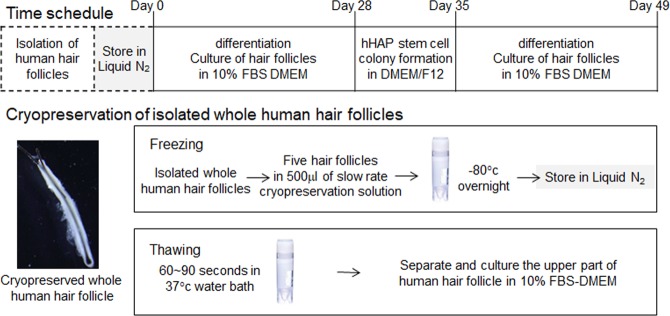
Protocol for hair follicle cryopreservation. A flow diagram is shown for procedures from hair follicle isolation, cryopreservation, thawing, upper-part of follicle culture, HAP-stem cell proliferation, and differentiation.

Cryopreservation of human whole hair follicles was done as previously reported^19,20^. Five whole hair follicles were extracted from the scalp and placed to cryovials^19,20^, followed by the addition of TC-Protector medium (DS Pharma Biomedical, Osaka, Japan). Cryovials were then kept overnight in a −80 °C freezer. After that they were placed in a liquid nitrogen tank. Stored cryovials containing whole hair follicles were thawed in a 37 °C water bath by gentle shaking. These thawed cells were divided in three fractions (upper, middle and lower). For inducing differentiation, the upper parts of hair follicles were used. The upper parts of hair follicles were dispersed in medium containing fresh DMEM (Sigma-Aldrich) with 10% FBS, 2 mM L-glutamine (Gibco), 10 mM Hepes (MP Biomedicals) and 50 µg/ml gentamycin (Gibco) in 6-well Corning Flat Bottom Cell Culture plates^20^ (Fig. 1).

### Immunofluorescence staining and flow cytometry analysis of the differentiated cells

Immunofluorescence staining was done as previously reported^19,20^. Immunostaining with primary antibodies for identification of differentiated cells and HAP stem cell colonies, for stem cell-markers are presented in Table 1. The following secondary antibodies were used: goat anti-mouse IgG and anti-mouse IgM, goat anti-rabbit IgG, goat anti-rat IgM, and donkey anti-goat IgG, conjugated to Alexa Fluor^®^ 568 or Alexa Fluor^®^ 594 (1:400; Molecular Probes). 4′, 6-diamino-2-phenylindole, dihydrochloride (DAPI) (Molecular Probes) was used to stain DNA.

**Table 1:** Primary antibodies used for immunofluorescence analysis.

Antibodies	Species	Source	Dilution	Used to identify
Nestin	Rabbit	Immuno-Biological Laboratories Co, Ltd (IBL), Gunma, Japan	1:50	Neurons
βIII-tubulin	Mouse	Covance, San Leandro, CA	1:500	Neurons
Glial fibrillary acidic protein (GFAP)	Mouse	Lab Vision, UK	1:200	Glial cells
GFAP	Chicken	Abcam, UK	1:300	Glial cells
keratin 15	Mouse	Lab Vision	1:200	Keratinocytes
Smooth muscle actin (SMA)	Mouse	Lab Vision	1:400	Smooth muscle cells
troponin (cTnT)-	Mouse	GeneTex, Taiwan	1:500	Cardiac muscle cells
SSEA1	Mouse	BioVision, Milpitas, CA	1:100	Stem cell
SSEA3	Rat	Millipore, Temecula, CA	1:100	Stem cell
SSEA4	Mouse	BioLegend, San Diego, CA	1:100	Stem cell
Nanog	Goat	R&D Systems, MN	1:100	Stem cell
Oct3/4	Goat	R&D Systems, MN	1:100	Stem cell

For flow cytometry analysis, the following secondary antibodies were used: goat anti-mouse IgG H&L phycoerythrin (1:500; Abcam), goat anti-chicken IgY biotinylated (1:500; R&D) antibodies, and Brilliant Violet 421^TM^ streptavidin (1:500; BioLegend).

### Real-time RT-PCR of stem cell marker genes

RT-PCR protocols to detect stem cell marker genes were performed as previously described^19^. Using RNeasy Plus Mini kit (QIAGEN), total RNA was extracted from 100 HAP stem cell colonies cultured from fresh and cryopreserved hair follicles. A high capacity RNA to cDNA kit (ABI) was used to synthesize c-DNA. CFX96 (Bio-Rad) with TaqMan Gene Expression Assays (ABI) was used to perform real-time PCR. The following TaqMan Probes were utilized: nestin: Hs00707120_s1, Oct3/4: Hs00999632_gl, Nanog: Hs02387400_g1, and r18s: Hs99999901_s1. Reference gene (r18s)^19^ was used to normalize the mRNA levels^19^.

### Western blotting

Western blotting was done as previously described^19^. Total proteins (30 μg/well) were extracted from HAP stem cell colonies cultured from fresh and cryopreserved hair follicles. Sodium dodecylsulfate polyacrylamide (4~20%) was used to perform electrophoresis. A immobilon-P membrane (Millipore Corporation) was used to transfer proteins. Specific protein on Immobilon-P were detected by rabbit polyclonal anti-nestin antibody (1:100), rabbit polyclonal anti-Oct4 antibody (1:500, Abcam), and rabbit polyclonal anti-GAPDH antibody (1:4000, BioLegend). The secondary antibody was peroxidase-conjugated protein A/G (Pierce). An enhanced chemiluminescence (ECL), together with a Western Blotting Detection System (Amersham Biosciences), were used to detect protein. ECL was measured by a Light-Capture CCD Camera System (ATTO, Tokyo, Japan).

### Statistical analysis

The experimental data represent the mean (±standard deviation, SD). Statistical analysis was conducted using an unpaired two-tailed students t-test. A p-value of <0.05 is considered statistically significant.

## Results

### Cryopreserved human hair follicles maintain the differentiation capacity of HAP stem cells

**Figure 2:**
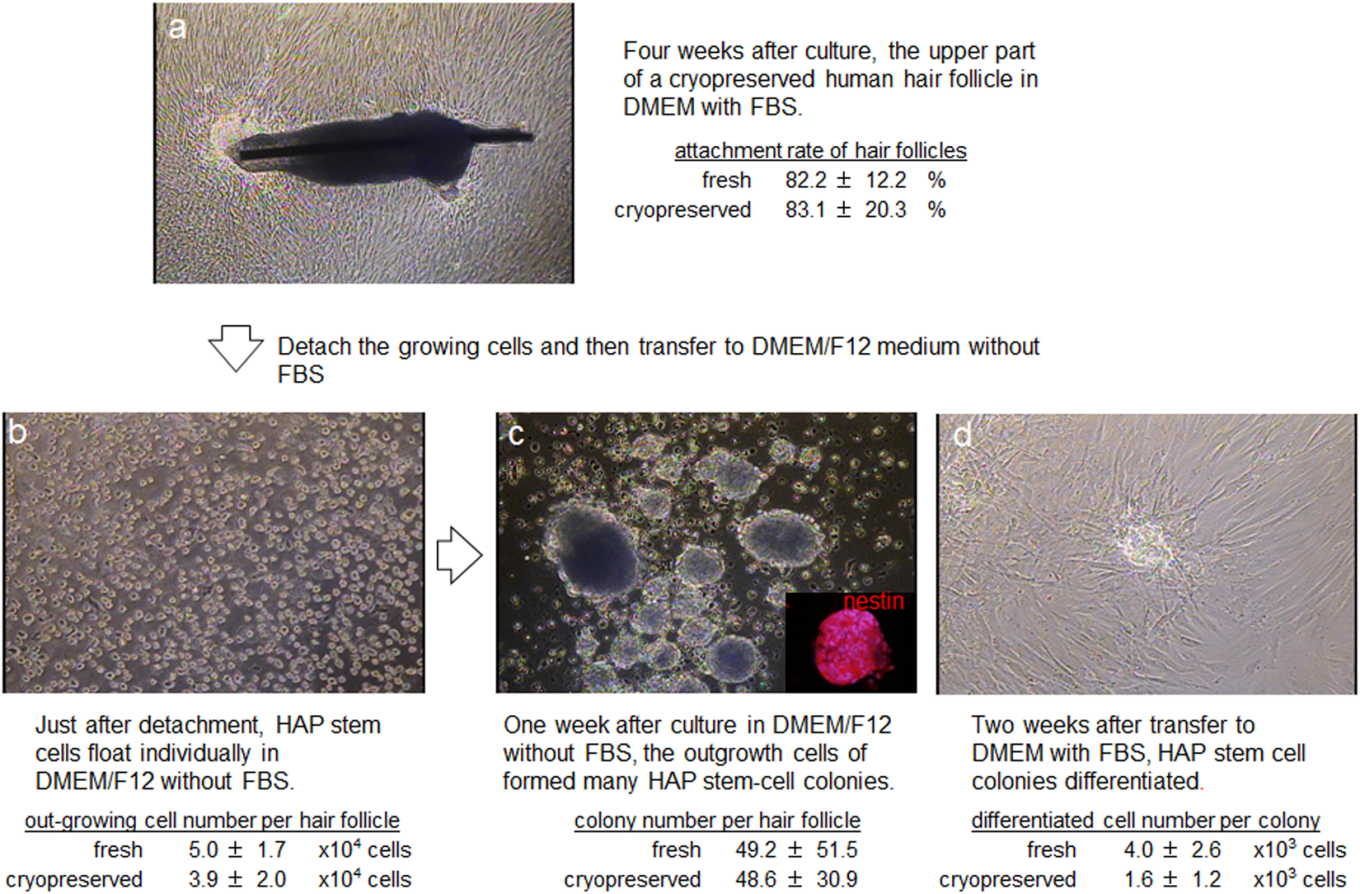
Images of a cultured cryopreserved human hair follicle and subsequent cell outgrowth.

To test the effect of cryopreservation on human hair follicles, we thawed and cultured them for four weeks in DMEM containing 10% FBS. Then the upper parts of the hair follicles were transferred to cell-culture dishes. The attachment rate of the hair follicles and numbers of proliferating cells were quantified. Fresh hair follicles (non-frozen control) showed an 82.2 ± 12.2% attachment rate and 5.0 ± 1.7 × 10^4^ growing cells/human hair follicle. Cryopreserved human hair follicles showed an 83.1 ± 20.3% attachment rate and 3.9 ± 2.0 × 10^4^ growing cells/ hair follicle. These results demonstrated that cryopreserved human hair follicles have the same outgrowth ability as fresh human hair follicles (Fig. 2a,b).

**Figure 3:**
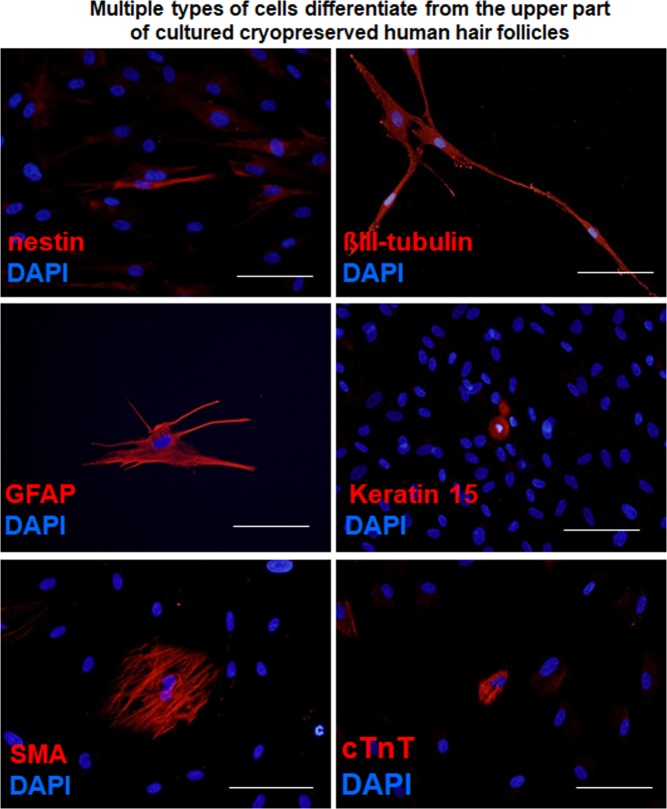
Cell types differentiated from the cultured upper part of cryopreserved hair follicles. Nestin- and βIII-tubulin-positive neurons, GFAP-positive glial cells, K15-positive keratinocytes, smooth muscle actin (SMA)-positive smooth muscle cells, and troponin (cTnT)-positive cardiac muscle cells. Staining for nestin, βIII-tubulin, GFAP, K15, SMA, and cTnT is shown in red color in corresponding panels. DAPI is shown in blue color in each panel. Scale bar = 100 µm.

After culturing the upper part of human hair follicles in DMEM containing 10% FBS for four weeks, human HAP stem cells proliferated and differentiated to nestin^+^ and βIII-tubulin^+^ neurons, GFAP^+^ glial cells, K15^+^ keratinocytes, SMA^+^ smooth-muscle cells, and cTnT^+^ cardiac muscle cells (Fig. 3). The following are the quantitative analysis of the differentiation ability of HAP stem cells: βIII-tubulin^+^ neurons, fresh 60.4 ± 16.6%, cryopreserved 55.1 ± 15.0%; SMA^+^ smooth muscle cells, fresh 18.3 ± 11.4%, cryopreserved 19.4 ± 11.0%; K15^+^ keratinocytes, fresh 5.2 ± 1.3%, cryopreserved 5.1 ± 3.1%; Troponin T^+^ cardiac muscle cells, fresh 2.2 ± 1.6%, cryopreserved 1.4 ± 1.5%; GFAP^+^ glial cells, fresh 7.3 ± 9.2%, cryopreserved 8.1 ± 9.8% (Table 2). These results show that HAP stem cells grown from cultured cryopreserved human hair follicles have a similar proliferation and pluripotent differentiation capacity as fresh human hair follicles.

**Table 2:** Cell types differentiated from HAP stem cells cultured from cryopreserved and fresh hair follicles.

	Fresh	Cryopreserved
***Four weeks culture of the upper part of human hair follicles***		
Neurons	60.4 ± 16.6%	55.1 ± 15.0%
Smooth-muscle cells	18.3 ± 11.4%	19.4 ± 11.0%
Keratinocytes	5.2 ± 1.3%	5.1 ± 3.1%
Cardiac-muscle cells	2.2 ± 1.6%	1.4 ± 1.5%
Glial cells	7.3 ± 9.2%	8.1 ± 9.8%
***Two weeks culture of HAP stem cell colonies***		
Neurons	76.3 ± 3.0%	67.8 ± 6.1%
Smooth-muscle cells	12.4 ± 5.2%	15.9 ± 3.7%
Keratinocytes	5.0 ± 3.7%	2.2 ± 1.5%
Cardiac-muscle cells	0.4 ± 0.4%	0.4 ± 0.5%
Glial cells	9.2 ± 11.3%	6.3 ± 5.4%

### Cultured cryopreserved human hair follicles produce HAP stem cell colonies

Outgrowing cells from the upper part of the cryopreserved hair follicle were also cultured for a week in DMEM/F12 medium without FBS containing B-27, and every two days were supplemented with basic fibroblast growth factor (bFGF). These cultured cells were able to produce similar number of HAP stem cell colonies as the upper part of fresh human hair follicles. HAP stem-cell colonies formed were: 49.2 ± 51.5 from fresh human hair follicles and 48.6 ± 30.9 from cryopreserved human hair follicles. These results show that cryopreserved human hair follicles have the same colony-forming ability as fresh human hair follicles (Fig. 2c).

**Figure 4:**
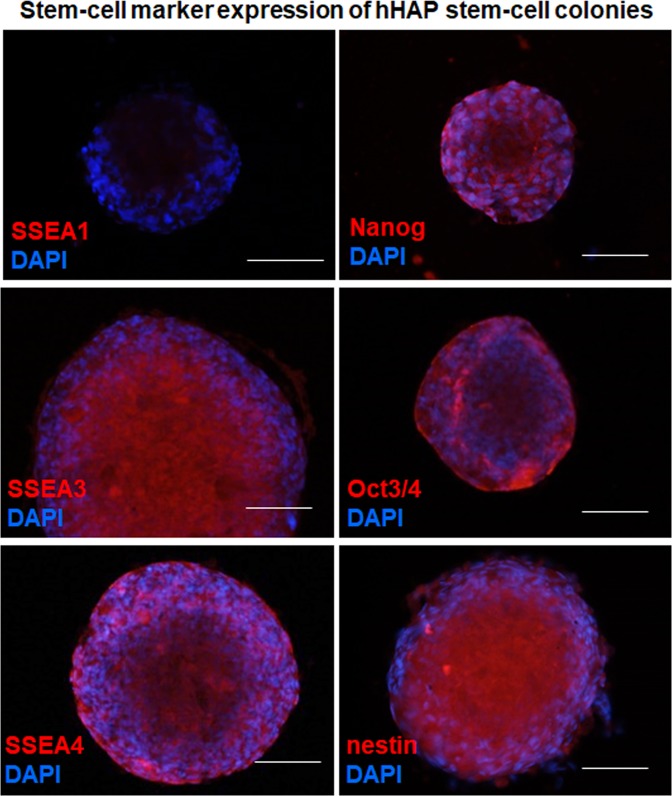
Stem-cell marker expression in HAP stem cell colonies produced from cultured cryopreserved human hair follicles. SSEA1-negative, SSEA3-positive, SSEA4-positive, Nanog-positive, Oct3/4-positive, and nestin-positive. Staining for SSEA1, SSEA3, SSEA4, Nanog, Oct3/4 and nestin is shown in red color in corresponding panels. DAPI is shown in blue color in each panel. Scale bar = 100 µm.

Immunofluorescence staining for stem cell marker expression in HAP stem cell colonies from cryopreserved hair follicles was performed on day 35 of culture, which showed SSEA1-negative (SSEA-1^−^), SSEA3-positive (SSEA-3^+^), SSEA4-positive (SSEA-4^+^), Nanog-positive, Oct3/4-positive, and nestin-positive (Fig. 4), which is similar to the HAP stem-cell colonies obtained from freshly-cultured human hair follicles^20^.

The HAP stem cell colonies that were obtained from the upper part of cryopreserved human hair follicle were cultured again back in DMEM with 10% FBS. After the switch into 10% FBS-containing DMEM for 2 weeks, the differentiating cells moved away from nestin-expressing HAP stem cell colonies and formed 4.0 ± 2.6 × 10^3^ differentiated cells/HAP-stem-cell colony. The HAP stem cell colonies that were obtained from the upper part of cultured cryopreserved human hair follicles formed 1.6 ± 1.2 × 10^3^ differentiated cells/HAP stem cell colony (Fig. 2d).

**Figure 5:**
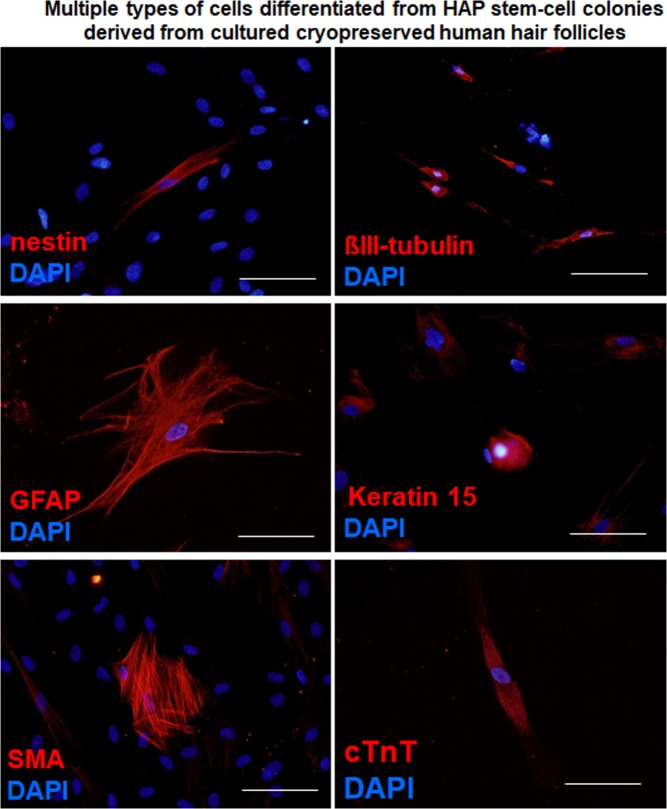
Cell differentiation from human HAP stem-cell colonies produced from cultured cryopreserved hair follicles. HAP stem cell colonies differentiated to nestin and βIII-tubulin-positive neurons, GFAP-positive glial cells, K15-positive keratinocytes, SMA-positive smooth muscle cells, and cTnT-positive cardiac muscle cells. Staining for nestin, βIII-tubulin, GFAP, K15, SMA, and cTnT is shown in red color in corresponding panels. DAPI is shown in blue color in each panel. Scale bar = 100 µm.

The cultured HAP stem cell colonies differentiated to nestin^+^ and βIII-tubulin^+^ neurons, GFAP^+^ glial cells, K15^+^ keratinocytes, SMA^+^ smooth muscle cells, and troponin^+^ cardiac muscle cells (Fig. 5). Immunofluorescence staining was performed on day 49. We then compared HAP stem cell colonies, produced from both the upper part of fresh human hair follicles and from the upper part of cryopreserved human hair follicles, for pluripotent differentiation capability. Both fresh and cryopreserved follicles had similar HAP stem-cell pluripotency and differentiating capacity as they produced multiple cell types: βIII-tubulin^+^ neurons, fresh 76.3 ± 3.0%, cryopreserved 67.8 ± 6.1%; SMA^+^ smooth muscle cells, fresh 12.4 ± 5.2%, cryopreserved 15.9 ± 3.7%; K15^+^ keratinocytes, fresh 5.0 ± 3.7%, cryopreserved 2.2 ± 1.5%; Troponin T^+^ cardiac muscle cells, fresh 0.4 ± 0.4%, cryopreserved 0.4 ± 0.5%; GFAP^+^ glial cells, fresh 9.2 ± 11.3%, cryopreserved 6.3 ± 5.4% (Table 2).

### Gene expression analysis in HAP stem cell colonies produced from cultured cryopreserved hair follicles compared to fresh hair follicles

**Figure 6:**
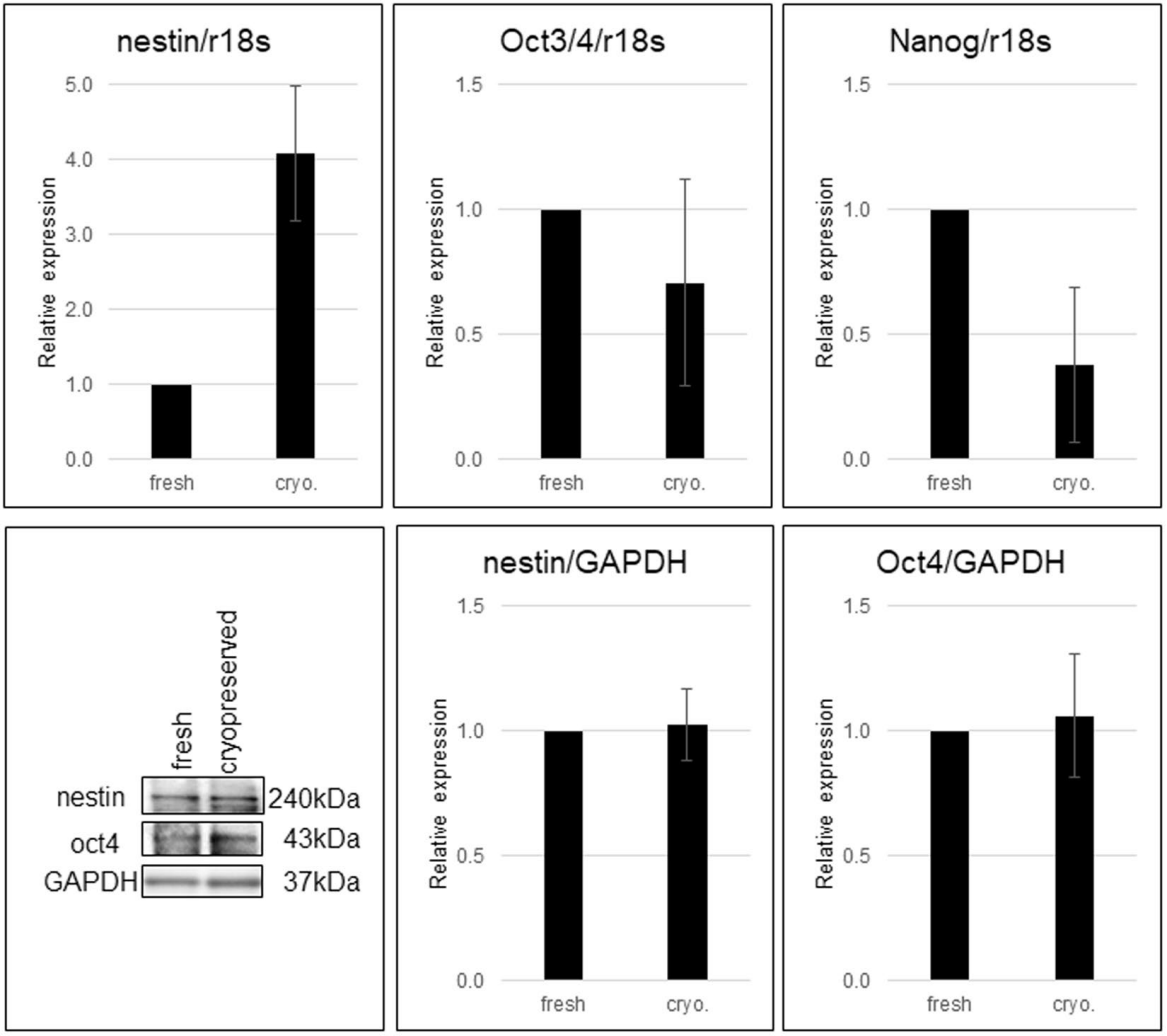
Gene expression in cryopreserved hair follicles compared to fresh hair follicles. The blots for nestin, Oct4 and GAPDH shown, in the lower left panel, are cropped from different gels (shown in Supplementary Information).

RT-PCR analysis (on day 35) showed the relative mRNA level of the HAP stem cell colonies formed from cultured cryopreserved hair follicles: for nestin 4.09 ± 0.90; for Oct3/4 0.71 ± 0.4; for Nanog 0.38 ± 0.31. The values are normalized to the expression of these genes for fresh fair follicles (Fig. 6, Supplementary Information). These results show that the mRNA levels of stem-cell marker genes such as nestin, Oct3/4, and Nanog were preserved in the HAP stem-cell colonies formed from cryopreserved human hair follicles.

The expression of nestin and Oct4 was compared by western blot analysis, between HAP stem cell colonies and cryopreserved hair follicles to fresh hair follicles. In comparison to HAP stem cell colonies from fresh hair follicles, the relative protein levels of HAP stem cell colonies formed from cryopreserved hair follicles were nestin 1.02 ± 0.14 and cultured Oct4 1.06 ± 0.25 (Fig. 6). These results show that HAP stem-cell colonies formed from cultured cryopreserved human hair follicles maintained normal protein levels.

## Discussion

The human hair follicle cryopreservation method presented in this study is suitable to produce large amount of HAP stem cells for future stem-cell-related regeneration medicine in the clinic. With this method, each individual will be able to obtain and bank their own HAP stem cells, which will be ideal because they will be free from immunological rejection as well as ethical or oncogenic issues compared to human oocytes, embryonic stem (ES) cells or induced pluripotent stem (iPS) cells.

The unique capacity of stem cells to produce various cell types is vital for regenerative medicine, drug screening, and tissue engineering. For efficient industrial-scale and clinical use of stem cells, vigorous and functional cryopreservation methods are required. Cryopreservation methods have been reported using ES cells, mesenchymal stem cells (MSCs), iPS and several somatic stem cells^21–40^. ES cells and iPS cells are sensitive to cryopreservation, which means they required specialized methods to maintain optimal cell viability and recovery^30^. Cryopreservation is also important to minimize metabolic activity, preserve viability, and differentiation potential of stem cells^31,32^. Zhao *et al*.^24^ reported that fetal human liver hematopoietic progenitor cells (LHSCs) can be cryopreserved for approximately two years with fully functional differentiation ability. Human dental pulp stem cells were shown to maintain proliferation and differentiation potential even after being stored in liquid nitrogen for two years^33^. Marquez-Curtis *et al*.^34^ demonstrated the ability of placental mesenchymal stem cells (MSCs) to differentiate after cryopreservation. In a study using bone marrow-MSCs (BM-MSCs), Shen *et al*.^35^ have shown that BM-MSCs retain multilineage differentiation ability even after 20 years of cryopreservation. Successful differentiation potential and viability was also found in cryopreserved adipose-derived stem cells (ADSCs)^36^. Hill *et al*.^37^ reported that cryopreservation does not affect the multipotency and differentiation capacity of human skin derived precursor cells (SDPCs). Lavoie *et al*.^38^ found no difference in growth potential of skin epithelial stem cells before and after cryopreservation. Previously, we showed that mouse whisker hair follicles when cryopreserved retained HAP stem cells and growth capacity after transplantation as well as viability of the HAP stem cells^39^. Gho *et al*.^40^ reported that cryopreserved human hair follicle bulge-derived stem cells that grew out from fresh follicles were able to maintain multilineage differentiation ability. Recently, several reliable long-term cryopreservation methods were developed for various kinds of stem cells with full preservation of multipotency^41–46^.

Here, we showed that the whole human hair follicle when cryopreserved by slow-rate cooling procedure maintains the pluripotency of their stem cells. The thawed follicles can be transplanted directly or cultured to produce HAP stem cell colonies for differentiation to nerve, glial, smooth muscle, cardiac, keratinocyte, and other cells. The cryopreservation method presented here will enable each individual to keep a bank of pluripotent stem cells from the cryopreserved hair follicles for future clinical application. The entire follicle can be cryopreserved without culture or other manipulation which makes this method potentially widely used.

## Supplementary information

supplementary information
