# Supplementary material for: Hair-follicle-associated pluripotent stem cells derived from cryopreserved intact human hair follicles sustain multilineage differentiation potential
Source: Sci Rep. 2019 Jun 27;9:9326. doi: 10.1038/s41598-019-45740-9 (PMC6597789; doi:10.1038/s41598-019-45740-9)
Supplement: Supplementary file 1 — supplementary information [file 41598_2019_45740_MOESM1_ESM.pdf]

Hair-follicle-associated pluripotent stem cells derived from cryopreserved intact human hair follicle sustain multilineage differentiation potential

Koya Obara, Natsuko Tohgi, Sumiyuki Mii, Yuko Hamada, Nobuko Arakawa, Ryoichi Aki, Shree Ram Singh, Robert M. Hoffman, and Yasuyuki Amoh

Full-length blots related to Figure 6

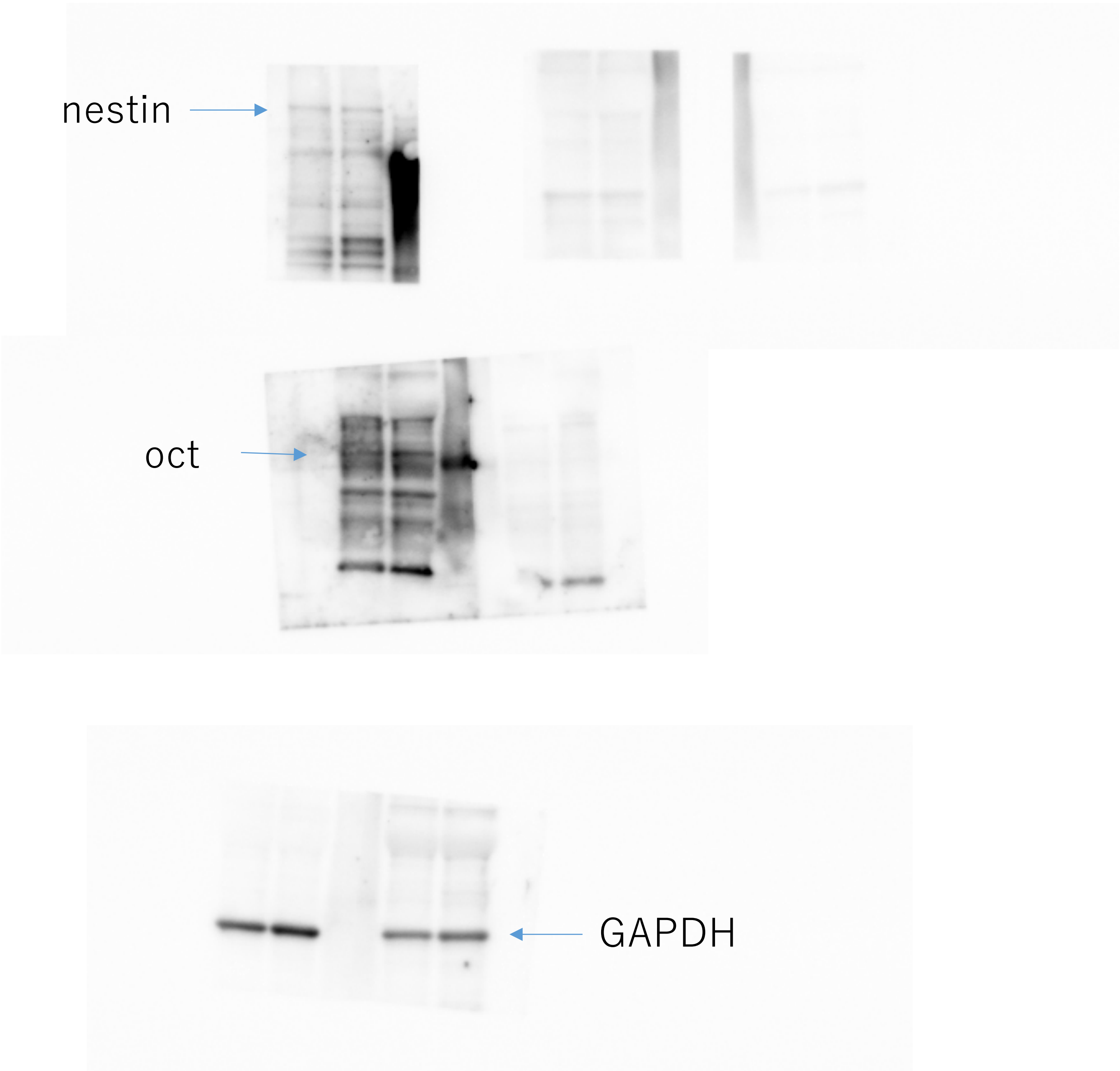
nestin
oct
GAPDH

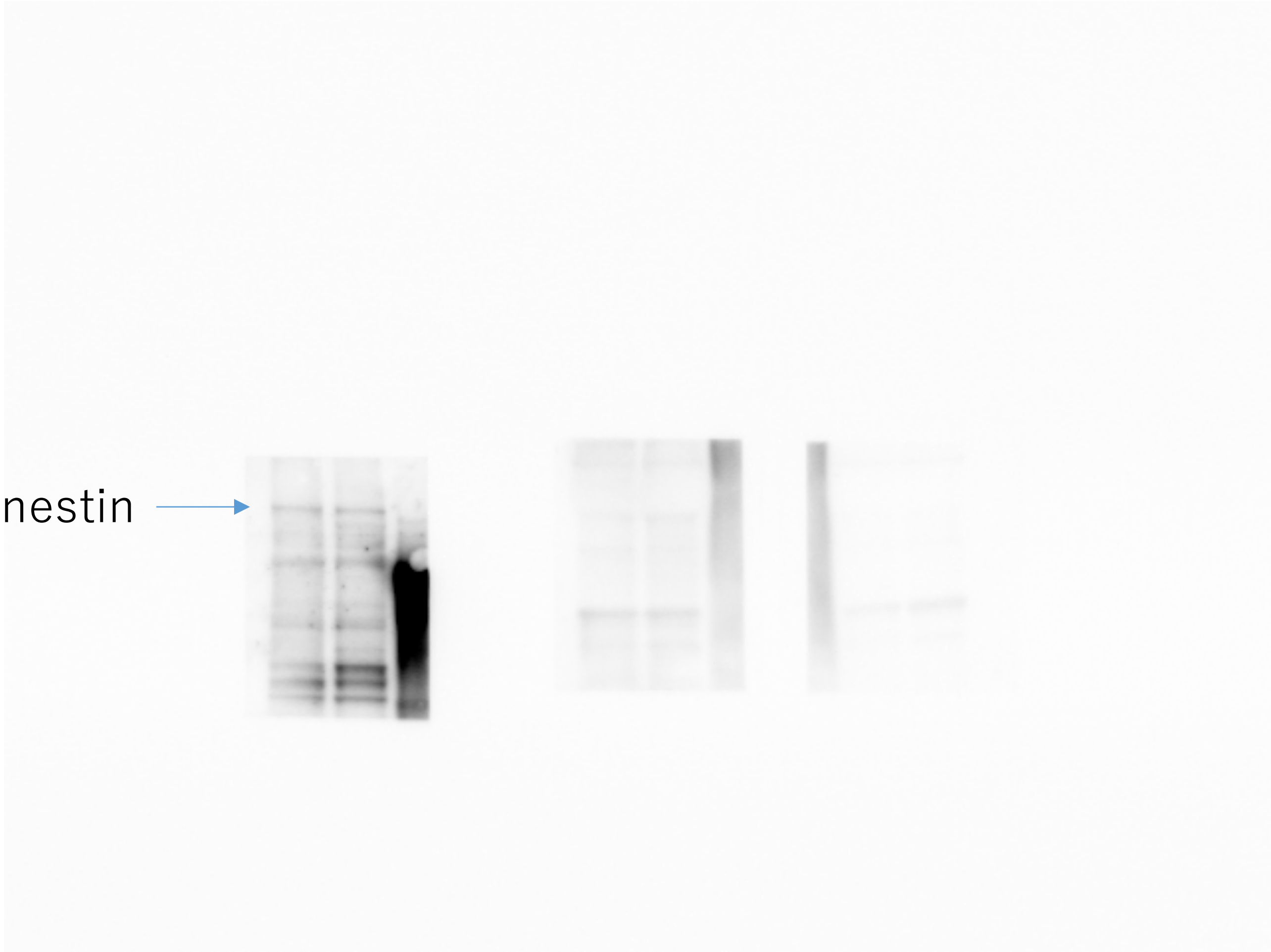
nestin

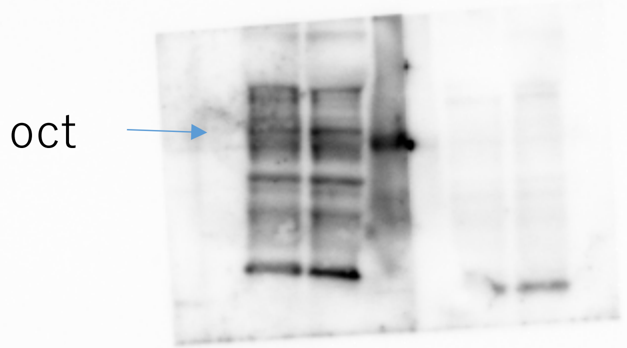
oct

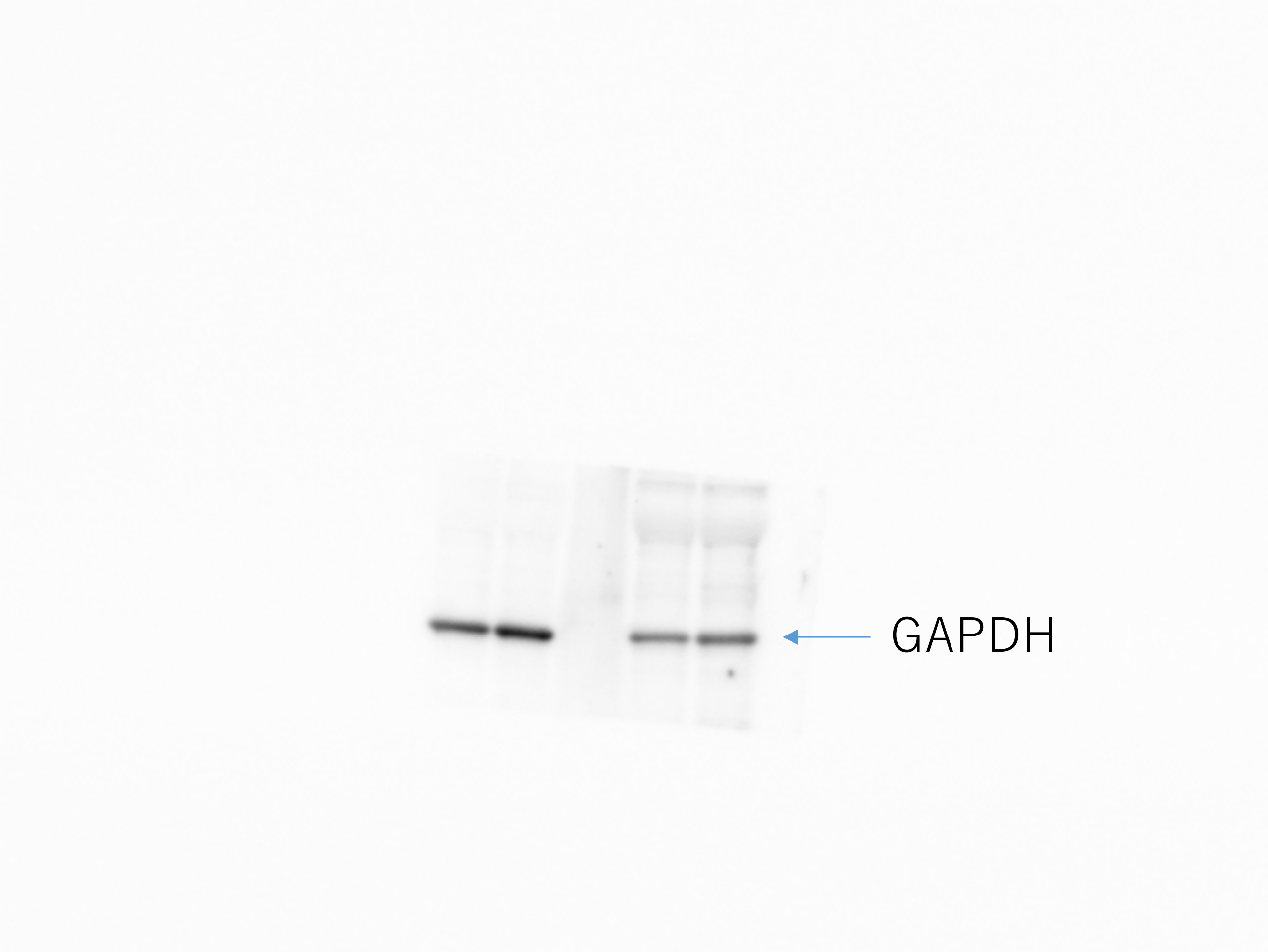
GAPDH
